# Data on before and after the Traceability System of Veterinary Antimicrobial Prescriptions in Small Animals at the University Veterinary Teaching Hospital of Naples

**DOI:** 10.3390/ani11030913

**Published:** 2021-03-23

**Authors:** Claudia Chirollo, Francesca Paola Nocera, Diego Piantedosi, Gerardo Fatone, Giovanni Della Valle, Luisa De Martino, Laura Cortese

**Affiliations:** Department of Veterinary Medicine and Animal Productions, University of Naples, “Federico II”, Via Delpino 1, 80137 Naples, Italy; claudia.chirollo@unina.it (C.C.); francescapaola.nocera@unina.it (F.P.N.); diego.piantedosi@unina.it (D.P.); gerardo.fatone@unina.it (G.F.); giovanni.dellavalle@unina.it (G.D.V.); laura.cortese@unina.it (L.C.)

**Keywords:** prudent use of antimicrobials, antimicrobial resistance, companion animals, veterinary medicine, electronic veterinary prescription

## Abstract

**Simple Summary:**

Veterinary electronic prescription (VEP) is mandatory by law, dated 20 November 2017, No. 167 (European Law 2017) Article 3, and has been implemented in Italy since April 2019. In this study, the consumption of antimicrobials before and after the mandatory use of VEP was analyzed at the Italian University Veterinary Teaching Hospital of Naples in order to understand how the traceability of antimicrobials influences veterinary prescriptions. The applicability and utility of VEP may present an effective surveillance strategy able to limit both the improper use of antimicrobials and the spread of multidrug-resistant pathogens, which have become a worrying threat both in veterinary and human medicine.

**Abstract:**

Over recent decades, antimicrobial resistance has been considered one of the most relevant issues of public health. The aim of our study was to evaluate the differences related to the prescription of antimicrobials at the University Veterinary Teaching Hospital, before and after the mandatory use of veterinary electronic prescription (VEP). In particular, the consumption of antimicrobials was examined, especially taking into consideration the recommendations of prudent use. A comparison of data collected before and after the use of electronic prescription highlighted that during the period chosen for the study, the choice of antimicrobial molecules was appropriate, favoring those of “first” and “second line.” However, prescription and the use of some molecules not registered for veterinary medicine were observed in the period before VEP. Broad-spectrum antimicrobials, including penicillins with *β*-lactamase inhibitors, as well as first-generation cephalosporins and fluoroquinolones, were the most frequently prescribed compounds. There are few studies conducted in Italy aimed at investigating the use of antimicrobials in companion animals under field conditions and with particular regard to prudent use recommendations. This type of study underlines the importance of electronic medical recording in veterinary practice and, above all, its usefulness in monitoring the use of certain antimicrobial agents classified as of critical importance in human medicine.

## 1. Introduction

In recent decades, antimicrobial resistance (AMR) has become an important issue worldwide, affecting many public health fields, such as human and veterinary medicine, animal husbandry, agriculture, the environment, and trade. Many studies show that direct contact with animals may, under certain conditions, play an important role in the transmission of resistance. This is potentially dangerous for people who live or work in close contact with animals, mostly livestock animals, but in recent years, cases in pets have also increased [1]. Antimicrobial resistance is currently a public health problem, and it is mainly linked to the inappropriate use of antimicrobials, both in humans and animals. Obviously, the risk exists when the same antimicrobial is used both in veterinary and human medicine or exhibits cross-resistance with other antibiotics used mainly in human medicine. Furthermore, a great concern is presented toward zoonotic-resistant bacteria, which can spread from animals to humans and vice versa, especially by direct contact with pets. Moreover, the appropriate use of antimicrobials to prevent AMR is not only important from a public health perspective but also for animal health and welfare [2]. In fact, the loss of the efficacy of antimicrobials, as found in human medicine, has also occurred in veterinary medicine [3,4]. In pets, antimicrobials are often prescribed for the treatment of various diseases. Generally, antimicrobial prescriptions are made to treat skin, respiratory, urinary, and gastrointestinal diseases [5]; however, over the years, the misuse and overuse of antimicrobials have led to the increased spread of multidrug-resistant bacteria among companion animals [6,7,8,9] for which there is often a lack of data on antimicrobials administration [10]. Furthermore, the consumption of antimicrobials differs among the different geographical areas. For instance, in Europe, broad-spectrum antimicrobials are widely used in small animal practice, and empirical antibiotic therapy is often administered [11]. In particular, *β*-lactams, such as amoxicillin and amoxicillin combined with clavulanic acid, are the most commonly used antimicrobials in dogs and cats in Norway, Finland, the United Kingdom, Switzerland, Italy, Belgium, and the Netherlands [12,13,14,15,16,17]. First-generation cephalosporins are also frequently dispensed, especially in dogs [18], and the increased use of third-generation cephalosporin cefovecin in cats has been reported in the UK [19]. The administration of lincosamides (clindamycin), fluoroquinolones, macrolides, tetracyclines (doxycycline), nitroimidazoles, and trimethoprim/sulphonamides has also been regularly described in small animal clinical settings, although to a lesser extent than *β*-lactams [20].

According to veterinary literature, antimicrobial therapy appears to be mainly empirical rather than based on antimicrobial susceptibility testing (AST). This finding presents an important issue mainly for the Critical Important Antimicrobial Agents (CIAs) such as fluoroquinolones, macrolides, and third- and fourth-generation cephalosporins. An Italian study highlighted that in a veterinary teaching hospital, less than 5% of antibiotic prescriptions were made following AST results [21]. This may be the reason why there has been an increasing number of guidelines for the prudent or responsible use of antimicrobials in dogs and cats, such as those suggested by the World Health Organization, the Federation of Veterinarians of Europe, and many other organizations. Moreover, since veterinary environments represent important “reservoirs” for multidrug-resistant bacteria and are the settings where indiscriminate antimicrobial use is displayed, veterinary hospitals are encouraged to develop and implement antimicrobial stewardship (AMS) programs, which include effective infection control, bacteriologic culture, and AST. The development of antimicrobial resistance can also be reduced by ensuring the proper prescription and traceability of antimicrobials.

In most cases, the lack of computerized prescriptions for veterinary medical products is one of the main problems in understanding antimicrobials, which are commonly used in each species and the relative amounts. However, some European countries have adopted specific action plans and also managed to provide precise information on drug prescriptions thanks to the computerization of most prescription practices.

Article 3 of Law No. 167 of 20 November 2017 called European Law [22], laying down provisions on the traceability of veterinary medicinal products and medicated feed for the achievement of the objectives of Directive 2001/82/EC [23], provided the adoption of a computerized system for the traceability of veterinary medicinal products and medicated feed through the introduction of veterinary electronic prescription (VEP). The new computerized system, implemented in Italy in April 2019, does not introduce any obligations or additional provisions compared to current rules (Legislative Decree No. 193/06 implementing an EU Directive of 2004) [24]. It replaced the paper form of the prescription on the whole national territory, simplifying procedures, reducing administrative obligations, improving control activities, and re-elaborating data useful to contain antimicrobial resistance.

The aims of the present study were: (i) to collect the prescription of clinicians at the University Veterinary Teaching Hospital of Naples, (ii) to analyze the clinicians’ preferential choices of antimicrobials, and (iii) to evaluate any differences in the prescriptions one year before and one year after the mandatory use of VEP.

## 2. Materials and Methods

### 2.1. Ethical Statement

The study’s protocol was approved by the Ethical Animal Care and Use Committee of the University of Naples Federico II (Certificate Number: PG/2021/0009256), in compliance with the Italian Legislative Decree 26/2014, Article 2.

### 2.2. Description of the Veterinary Teaching Hospital

The Department of Veterinary Medicine and Animal Productions (University of Naples Federico II, Naples, Italy) hosts a University Veterinary Teaching Hospital called Ospedale Veterinario Universitario Didattico (OVUD) that is divided into several units (Internal Medicine, Surgery, Imaging, Animal Reproduction, 24 h Emergency Service, and Intensive Care Unit). Activities carried out in the hospital include diagnostic, clinical, surgical, and preventive practices on small and large animals. In addition, OVUD offers a one-year rotating clinical internship program in small animal medicine and surgery. Thirty vets (professors, researchers, and Ph.D. students) participate in the above OVUD professional activities, including students who perform a training period before graduation. OVUD is connected to different diagnostic support services such as hematology and clinical biochemistry, parasitology, microbiology, genetics, histocytopathology, and necropsy.

### 2.3. Data Collection

The study was conducted with a retrospective cross-sectional design. All data concerning the antimicrobial veterinary prescriptions related to the period of study before the introduction of VEP (from April 2018 to March 2019) were collected by software called “Pongo^®^” (TELE.MA.CO, via Savona 29, Rome, Italy), a database that holds information on the activity of the different OVUD units. Furthermore, all data concerning the antimicrobial veterinary prescriptions related to the period of study after the introduction of VEP (from April 2019 to March 2020) were collected by a new computerized system called “VET INFO,” an internet portal of the Ministry of Health, where each veterinarian enters data and issues the recipe (www.vetinfo.sanita.it; accessed on 15 December 2020).

The study population included client-owned dogs and cats referred daily to the hospital. The medical record of each dog and cat associated with antimicrobial drug prescription was reviewed, and the condition for which the patient received antimicrobial drugs was categorized as: skin, respiratory, gastrointestinal, genitourinary, odontostomatologic, ophthalmologic, ear, orthopedic diseases, surgical procedures (neurosurgery, soft tissues, and oncological surgery), and recovery ward. Data that did not belong to these categories were classified as “other.”

### 2.4. Data Management and Statistical Analysis

The collected data were initially recorded using spreadsheet software (Microsoft^®^ Excel^®^ 2011) and then imported into a software package (JMP version 9, SAS Institute, New York, NY, USA) used to perform the statistical analysis. The Chi-square (Χ^2^) nonparametric test was performed to determine the independence between antimicrobial prescription and the use of VEP, for dog and cat species, respectively.

## 3. Results

### 3.1. Antimicrobial Prescription before VEP

A total of 1149 drug prescriptions was detected in the Pongo database starting one year before the mandatory use of VEP. Of these, 41.6% belonged to antimicrobials (No. 478) and contained one single-active compound (370/478; 77.4%), while the remaining 22.6% (108/478) combined two molecules (amoxicillin/clavulanic acid, metronidazole/spiramycin, and benzylpenicillin/dihydrostreptomycin).

Several molecules with antimicrobial activity were used in dogs and cats referred to the hospital during this first study period (Table 1), with *β*-lactams, alone or combined, (268/478; 56.1%) as the most widely used antimicrobial class, followed by imidazole derivatives, alone or combined, (60/478; 12.6%), fluoroquinolones (45/478; 9.5%), and aminoglycosides (34/478; 7.1%).

**Figure 1:**
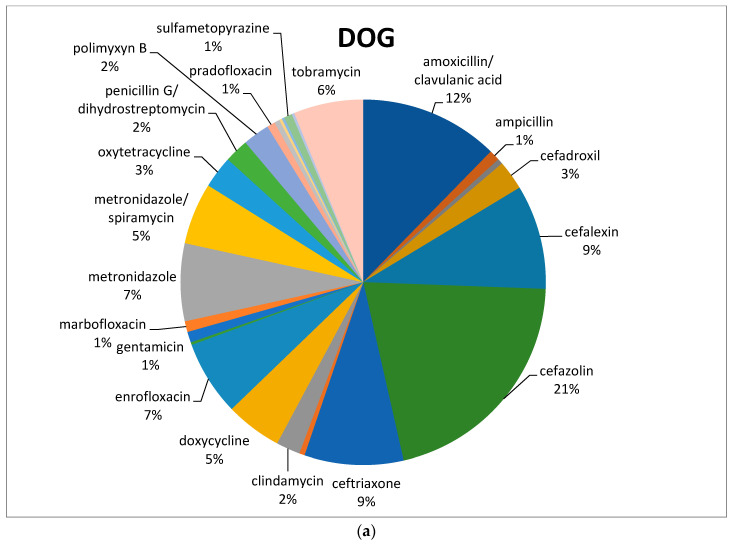

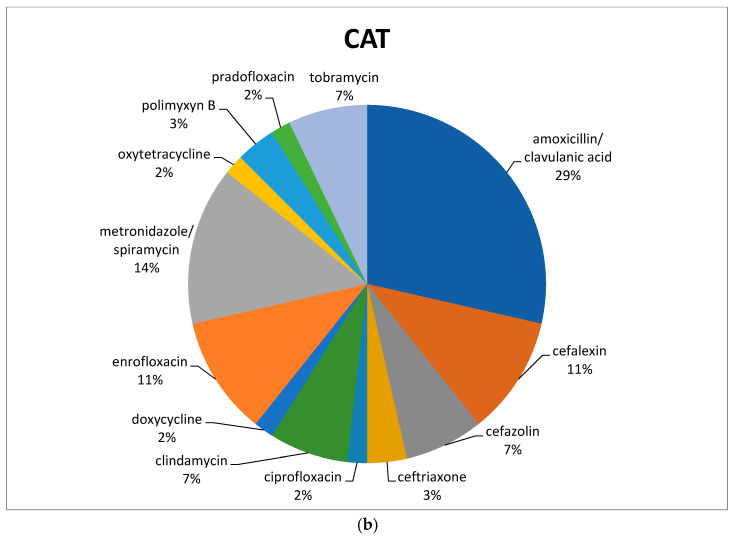
Percentage of selected antimicrobials prescribed in dogs (**a**) and cats (**b**) before the mandatory use of VEP.

Of the active principles, cefazolin (88/422; 21%) was the most common antimicrobial used in dogs, followed by amoxicillin/clavulanic acid (52/422; 12%), metronidazole (alone or in combination with spiramycin) (52/422; 12%), cephalexin (39/422; 9%), ceftriaxone (37/422; 9%), and enrofloxacin (28/422; 7%) (Figure 1a).

In feline species, the most commonly prescribed antimicrobial was amoxicillin/clavulanic acid (16/56; 29%), followed by metronidazole in association with spiramycin (8/56; 14%). Both cefalexin and enrofloxacin reached 11% (Figure 1b), with a total of 6/56 prescriptions each (Table 1).

Regarding the route of administration, the distribution of antimicrobial treatments showed that most (430/478; 90%) fell into systemic use (oral or parenteral) and the remaining 10% (No. 49) into topical application (skin, eye, and ear). *β*-lactams, macrolides, lincosamides, tetracyclines, sulphonamides, fluoroquinolones, and metronidazole were always used systemically, whereas polymyxins and fusidic acid were only used topically. Aminoglycosides were both used topically (gentamicin and tobramycin) and parenterally (dihydrostreptomycin).

Three of the identified antimicrobials (ciprofloxacin, cefazolin, and ceftriaxone), even if not authorized for veterinary use in Italy, were used in off-label treatment. The oral fluoroquinolone ciprofloxacin was prescribed both in dogs and cats to treat urinary infections, while the cefazolin and ceftriaxone, first- and third-generation cephalosporins, respectively, were mainly used in hospitalized patients.

**Figure 2:**
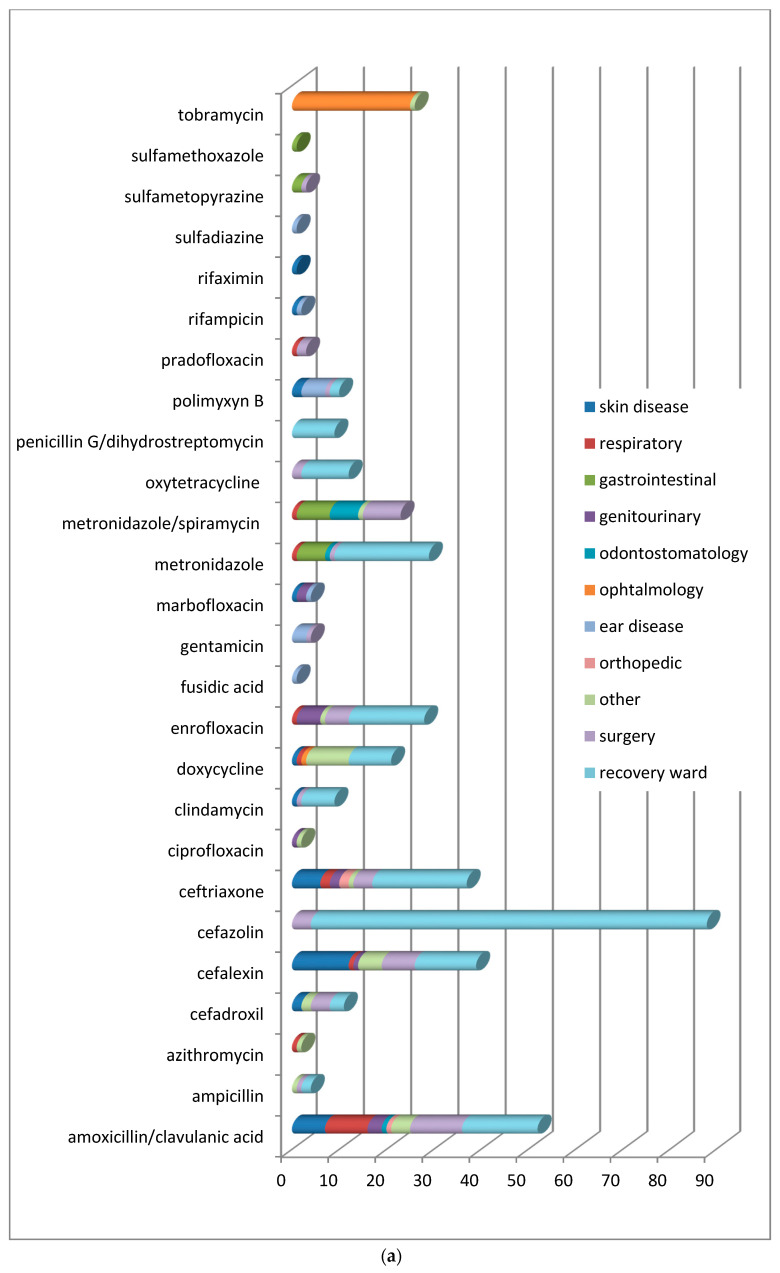

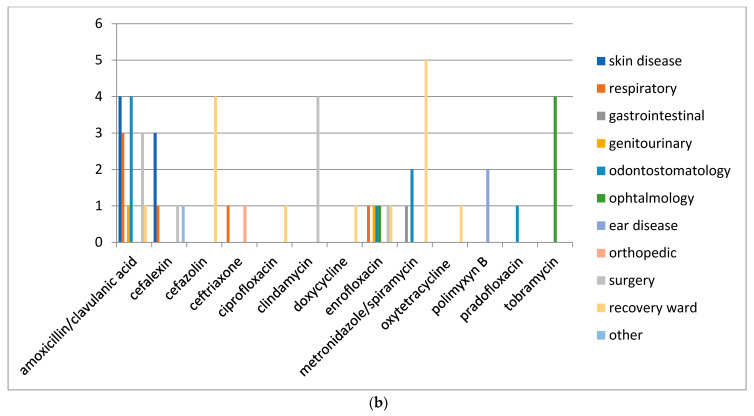
(**a**) Distribution of No. 422 antimicrobials treatments according to the organ/system in dogs before VEP. (**b**) Distribution of No. 56 antimicrobials treatments according to the organ/system in cats before VEP.

In order to understand which molecules were mostly used to treat infections involving specific organic systems, Figure 2a,b indicates the class of antimicrobials or the active compounds preferred both in dogs and cats. The results showed that first-generation cephalosporins (cephalexin) and amoxicillin/clavulanic acid were the molecules most used in pets suffering from skin infections. Local therapies with chlorhexidine and EDTA-based detergents were preferred in the treatment of external otitis, while the topical administration of antimicrobials such as rifampicin, polymyxin B, marbofloxacin, and gentamicin, was prescribed to treat complicated and chronic cases of otitis media.

In ocular problems and especially in conjunctivitis, antimicrobials for topical use based on tobramycin were used, and only in one complicated case was systemic doxycycline prescribed. In respiratory tract infections, the most commonly prescribed therapy, both in dogs and cats, was amoxicillin/clavulanic acid.

In infections of the oral cavity, the use of amoxicillin/clavulanic acid prevailed, followed by the administration of metronidazole alone or in combination with spiramycin.

In acute gastrointestinal diseases, the most widely used active ingredients were metronidazole and sulfonamides.

For the treatment of genitourinary tract infections, enrofloxacin, followed by amoxicillin/clavulanate, were the antimicrobials usually prescribed. Ceftriaxone was the antimicrobial of choice to handle orthopedic problems resulting from fractures.

Included in the section named “other” were all the pathologies deriving from traumatic events and some tick-borne infections requiring antibiotic therapy (*Ehrlichiosis*,* Lyme disease*). The only differences found in dogs and cats (Figure 2b) concern the choice of molecules in neurosurgery, soft tissue and oncological surgical procedures, and during hospitalization. In the canine species, the antimicrobials more frequently used were amoxicillin/clavulanic acid, cephalexin, metronidazole/spiramycin for surgery, cefazolin, ceftriaxone, and metronidazole during hospitalization. In the feline species, clindamycin and amoxicillin/clavulanic acid were generally used for surgery, while cefazolin and metronidazole/spiramycin were administered in the recovery ward.

### 3.2. Antimicrobial Prescription after VEP

From April 19, 2019, the computerized database of the veterinary hospital was integrated by VEP. A total of 1182 drug prescriptions were examined, and 428 antimicrobials were detected (36%). Half of the electronic prescriptions were referred to as a single antimicrobial (216/428; 50%) and the remaining 212/428 to the combination of two active principles (amoxicillin/clavulanic acid, metronidazole/spiramycin, and benzylpenicillin/dihydrostreptomycin). Among the prescribed antimicrobial classes (Table 2), *β*-lactams, alone or in combination (59.3%), were the most widely used, followed by fluoroquinolones (47/428; 11.1%), nitroimidazoles (43/428; 10%), and aminoglycosides (33/428; 7.7%). As shown in Table 2, all other drug classes each represented <5% of total prescriptions.

The antimicrobials prescribed before and after VEP were compared, and there were only significant differences (*p* < 0.05) for some antimicrobials administered to dogs, precisely amoxicillin/clavulanic acid, cefadroxil, cefalexin, fusidic acid, gentamicin, marbofloxacin, metronidazole, metronidazole/spiramycin, and oxytetracycline, whereas no significant difference (*p* > 0.05) for antimicrobial prescriptions’ pre- and post-use of VEP for cats was observed.

Results on the administration route, after VEP, showed that most of the antimicrobials (88%) fell into systemic use (oral or parenteral), while for the remaining 12%, the topical use was preferred (skin, eye, and ear).

**Figure 3:**
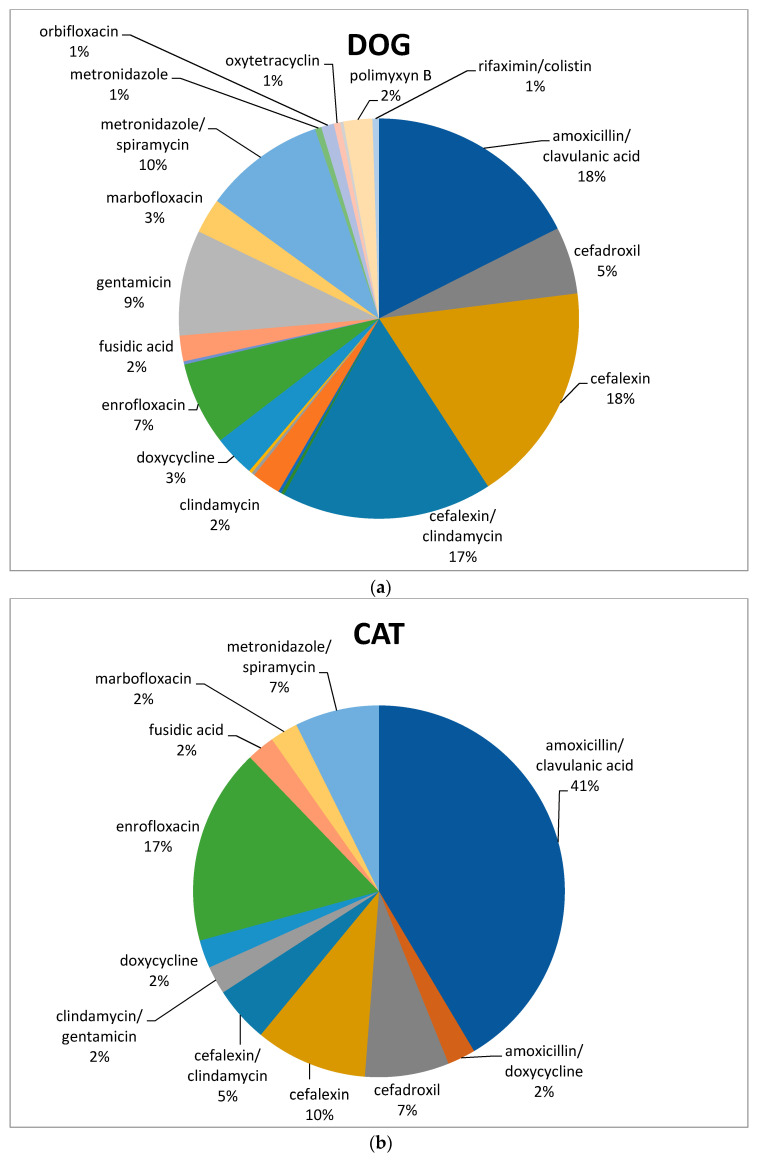
Percentage of selected antimicrobials prescribed in dogs (**a**) and cats (**b**) after the mandatory use of VEP.

In dogs, both cefalexin and amoxicillin/clavulanate were the molecules most frequently used (68 and 69/387, respectively; 18%), followed by association of cephalexin and clindamycin (66/387; 17%), metronidazole/spiramycin (38/387; 10%), gentamicin (33/387; 9%), and enrofloxacin (26/387; 7%) (Figure 3a). Amoxicillin/clavulanic acid was the most frequently prescribed antimicrobial in cats (17/41; 41%), followed by enrofloxacin (7/41; 17%) and cefalexin (4/41; 10%) (Figure 3b).

As far as clinical conditions were concerned, antimicrobials were prescribed to treat infections affecting the skin, eye, ear, oral cavity, gastrointestinal tract, and genitourinary and respiratory tract diseases. The surgery section included neurosurgery and soft tissue and oncological procedures, and hospitalized patients were included in the recovery ward.

**Figure 4:**
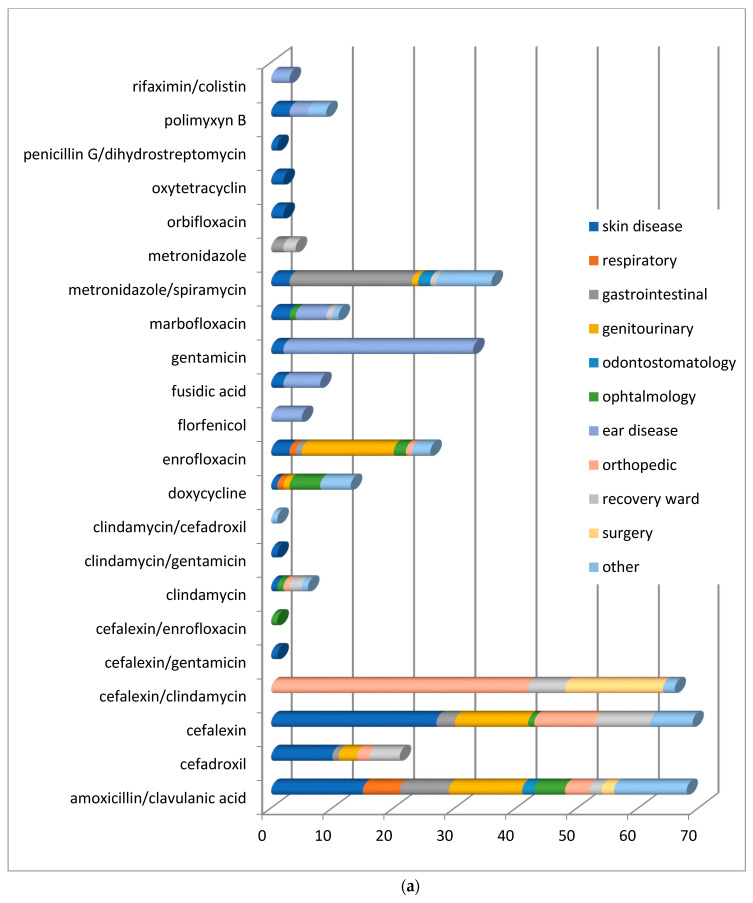

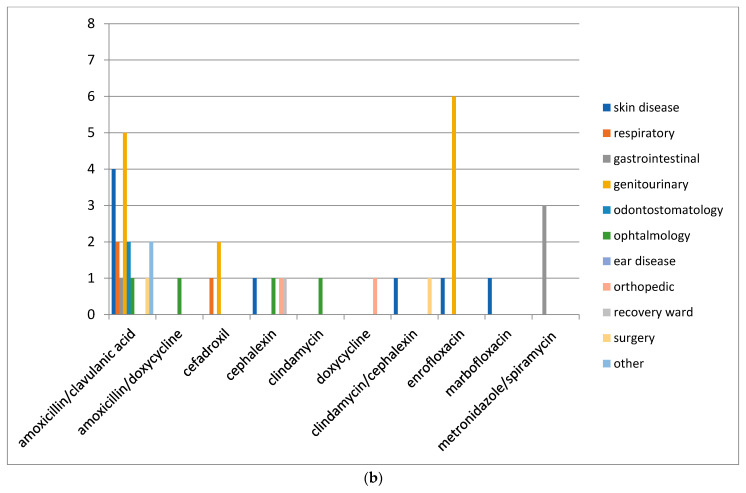
(**a**) Distribution of No. 387 antimicrobial treatments according to the organ/system (conditions) in dogs after the mandatory use of VEP. (**b**) Distribution of No. 41 antimicrobials treatments according to the organ/system (conditions) in cats after the mandatory use of VEP (from April 2019 to March 2020).

The distribution of antimicrobial compounds used for the treatment of conditions affecting the different organs/systems is reported in Figure 4a,b.

The treatment of skin and genitourinary tract infections of dogs and cats was performed using first-generation cephalosporins and fluoroquinolones, respectively. The treatment of gastrointestinal diseases involved penicillins with *β*-lactamase inhibitors, spiramycin/metronidazole, and fluoroquinolones.

In respiratory tract infections, the most regularly prescribed antimicrobial, both in dogs and cats, was amoxicillin in association with clavulanic acid.

In orthopedic diseases, an association of cefalexin and clindamycin was preferred, while in affections of the oral cavity prevailed the use of amoxicillin in association with clavulanic acid and metronidazole alone or in combination with spiramycin.

## 4. Discussion

### 4.1. Antimicrobial Prescription before VEP

Almost 41.6% of drug prescriptions collected from the OVUD database belonged to antimicrobials. In agreement with previous studies, we found that *β*-lactams were the most frequently prescribed class, followed by imidazole derivatives, aminoglycosides, and fluoroquinolones. In fact, as already mentioned in Section 1, *β*-lactams, such as amoxicillin and amoxicillin combined with clavulanic acid, have been the most commonly used antimicrobials for dogs and cats in many European countries (Norway, Finland, the United Kingdom, Switzerland, Italy, Belgium, and the Netherlands) [12,13,14,15,16,17]. First-generation cephalosporins were also frequently dispended, especially in dogs [18]. The administration of lincosamides, fluoroquinolones, macrolides, tetracyclines, nitroimidazoles, and trimethoprim/sulphonamides has also been regularly described in small animal clinical settings, although to a lesser extent than *β*-lactams [20]. Therefore, as well as in our study, in Europe, the prescription’s tendency was the wide use of broad-spectrum antimicrobials in small animal practice and frequent recourse to empirical antibiotic therapy [11].

Before VEP, in canine species, cefazolin was the most common antimicrobial used, followed by amoxicillin with clavulanic acid, metronidazole (often in association with spiramycin), cephalexin, ceftriaxone, and enrofloxacin. The most commonly prescribed antimicrobial in cats was amoxicillin/clavulanic acid, followed by metronidazole/spiramycin, cefalexin, and enrofloxacin.

The first-line agent amoxicillin/clavulanate is a broad-spectrum inexpensive drug with few side effects, and its use as the first choice for suspected infections without culture and sensitivity testing is approved by most of the available guidelines. Cefazolin/cephalexin, like amoxicillin/clavulanate, was used in a high proportion of patients without a culture. According to our results, the laboratory diagnosis to correctly identify the bacterial agent and assess its susceptibility to antimicrobials resulted in an almost unused tool. Escher et al. [21] first conducted a study in the veterinary teaching hospital of the University of Pisa (Italy) and reported that not even 5% of the antimicrobials prescribed for pets were supported by susceptibility testing. In another research conducted in Italy in 2005, laboratory diagnosis was performed before treatment only in 12% of gastroenteritis and 41% and 50% of pyoderma and urinary tract infections, respectively [25]. In a survey on antimicrobial prescribing patterns in small animal veterinary clinics of Emilia Romagna Region (Italy), only 7% of clinicians had the habit of always waiting for AST results before starting treatment [26].

The empirical use of antimicrobials should always be avoided, and the causal infectious agent and its susceptibility to the active substance should be ascertained before starting antimicrobial therapy. In particular, an antibiotic sensitivity test should be always performed for recurrent infections, such as urinary or skin infections, where the rearing of resistant microorganisms is favored. However, only in a few cases (serious illness or outbreak of a bacterial infection with high mortality or rapid spread) may antimicrobials be initiated on the basis of clinical diagnosis [27].

Some prescriptions concerning first-generation cephalosporins, such as cefazolin and cephalexin, were, for “unknown target infection,” followed by a wound or abscess. These antimicrobials are generally used as relatively narrow-spectrum antimicrobials under current guidelines.

Based on the collected data, veterinarians, before the mandatory use of VEP, did not frequently prescribe combinations of different antimicrobials (24%). In fact, most of them preferred the choice of products containing one single-active principle (76%).

Our data revealed the use of three antimicrobials not authorized for veterinary medicine in Italy. The first was the fluoroquinolone ciprofloxacin, usually used as an alternative to enrofloxacin, for urinary infections treatment both in dogs and cats. The other two were injectable cephalosporins, cefazolin and ceftriaxone. Cefazolin was used for perioperative prophylaxis and ceftriaxone during hospitalization in dogs and cats. Use deviating from the Summary of Product Characteristics (SPC), also called off-label use, is not permitted by Dlsg 193/06, except in particular cases. The off-label utilization of cefazolin and ceftriaxone as injectable products for human use only was due to the availability of injectable cephalosporins registered for veterinary use only for large animals (livestock animals) in Italy. The extent of the off-label use of critically important antimicrobials (CIAs) for human medicine is an under-investigated area. The off-label use of antimicrobials licensed for human beings in animals should be exceptional, under the professional responsibility of the veterinarian, and limited to cases where no other suitable products are available for that species or pathology (Dlgs 193/06). In pets, application via the cascade system of medicinal products for human use, and often of medicinal products that are only authorized for use in humans, requires extremely careful assessment and a well-founded risk–benefit analysis.

### 4.2. Antimicrobial Prescription after VEP

Analyses of the prescriptions obtained through the new electronic traceability system showed a lower percentage of antimicrobials compared to the previous database. Only 36% of drugs employed in the OVUD belonged to antimicrobials, but nearly half of the computerized prescription contained two active principles. The decrease of antimicrobials after the implementation of VEP could be explained by analyzing different factors. Italy has been one of the first European countries where VEP came into force. The new traceability system is under the direct control of the Ministry of Health through the VET INFO portal. Veterinary electronic prescription is issued by a tablet, computer, or mobile application and completely replaces the paper form. Data regarding treatments are quickly consulted by competent authorities. In fact, Italian veterinarians’ prescribing behavior is now under the control of the Ministry of Health, especially for antimicrobial prescriptions. In addition, the increase of new European regulations, guidelines, and stewardship programs to counteract the AMR has implemented the consciousness in the choice of the correct antimicrobial therapy among veterinarians.

The most frequent associations were metronidazole/spiramycin and cephalexin/clindamycin. The combination of different antimicrobials could be indicated in specific cases to obtain a synergistic effect, to allow lower doses of either active ingredients, or to prevent the emergence of resistance, but trying to respect an appropriate choice of four-quadrant coverage [28,29]. However, this strategy has not been implemented due to cost concerns and the potential enhanced toxicity derived from the use of more than one agent. In addition, the mixture of antimicrobials with different mechanisms of action can even be antagonistic or inefficacious. It would be necessary to avoid using combination therapy unless there is clearly a life-threatening infection present and/or an unpredictable antimicrobial susceptibility of the pathogen(s) involved. In particular, the frequent empirical prescription performed in OVUD of a combination of cephalexin and clindamycin does not appear an appropriate choice of four-quadrant coverage because both molecules have the same spectrum of action. Finally, a bactericidal antibiotic (cephalexin) should not be combined with a bacteriostatic antibiotic (clindamycin).

After VEP, treatments with *β*-lactam antimicrobials were the most widely prescribed, followed by fluoroquinolones, nitroimidazoles, and lincosamides. Nitroimidazoles were frequently prescribed in our hospital for gastrointestinal diseases. It is noteworthy that the frequent empirical prescription of metronidazole (nitroimidazole) alone or in combination with spiramycin, for the treatment of gastrointestinal diseases, should be discouraged because of a significant short-term impact on the microbiome [30] and for its neurotoxicity [31]. In addition, clinicians should consider using antimicrobials in gastrointestinal diseases only after appropriate dietary trials have been unsuccessful and only after histopathologic evaluation of gastrointestinal biopsies in all cases in which it is possible [32]. Therefore, the use of antimicrobials should be reserved for patients in whom all other conditions are excluded and other empirical treatments have been exhausted.

Concerning our data in dogs after VEP use, large use of cefalexin and amoxicillin/clavulanate, followed by the association of cephalexin and clindamycin, metronidazole/spiramycin, gentamicin, and enrofloxacin, was found. Clavulanic-acid-potentiated amoxicillin was the most habitually prescribed antimicrobial in cats, followed by enrofloxacin and cefalexin.

In addition, after VEP off-label use or the use of high-importance antimicrobials that are not registered for veterinary medicine was not found in our data. This finding is in line with European recommendations of the correct use of antimicrobials (Commission Notice: Guidelines For the Prudent Use of Antimicrobials in Veterinary Medicine (2015/C 299/04)). In fact, a number of compounds on the World Health Organization’s list of critically important antimicrobials [33] are only authorized in medicinal products for human use. As laid down in EU legislation (Directive 2001/82/EC of the European Parliament and of the Council of 6 November 2001), those that do not have marketing authorization as veterinary medicinal products for use in food-producing animals may only be used off-label (following the cascade) in these animals if the substance in question is listed in Table 1 of the Annex to Commission Regulation (EU) No 37/2010 [34]. Off-label use (cascade) of the compounds referred to above for non-food-producing animals (e.g., pets and horses not intended for food production) should be avoided and strictly limited to very exceptional cases, where there are ethical reasons for doing so, and only when laboratory antimicrobial susceptibility tests have confirmed that no other antimicrobial would be effective.

Regarding the prescriptions, before and after VEP, significant differences were detected for some antimicrobial prescriptions in dogs, whereas no significant difference for cats was observed. In particular, the increasing use, in dogs, of first-generation cephalosporin cefalexin after the mandatory use of VEP could be related to the reduced prescription of third-generation cephalosporins, such as ceftriaxone, which is registered only for human use. Local treatments based on gentamicin and fusidic acid were preferred for skin and ear diseases compared to systemic therapy with other molecules. Doxycycline is mainly utilized in our geographical area for the therapy of many tick-borne diseases, such as Ehrlichiosis. Thus, the decrease of this compound could depend on a reduction of the incidence of these diseases, probably linked to the greater use of antiparasitic drugs. Moreover, after VEP, metronidazole alone was almost completely replaced by the combination of spiramycin/metronidazole.

Finally, it was not possible to compare our data after the introduction of VEP with data from other European countries because Italy is one of the few European countries that has replaced the paper form with the electronic prescription, and we did not find veterinary literature of similar reports.

## 5. Conclusions

Antimicrobial prescription data collected before and after the mandatory use of VEP suggest that broad-spectrum antimicrobials such as amoxicillin/clavulanate and first-generation cephalosporins are mainly used in dogs and cats. The antimicrobial prescriptions in our hospital are quite similar to those previously described in several European countries. In addition, this prescriptive trend is in perfect harmony with the recent categorization of antimicrobials in the European Union for prudent and responsible use in animals [35].

The only critical issues remain linked to the consumption of drugs authorized only in humans such as ceftriaxone, even if it disappeared after the introduction of VEP, and the empirical use of an antimicrobial combination. Increased use of AST could reduce the empirical prescription of broad-spectrum antimicrobials, encouraging other molecules that are equally effective but less risky to public health.

The mandatory nature of the electronic prescription requires validation over time and also to improve its functions and obtain useful data to combat antibiotic resistance. However, VEP may be considered as one more national surveillance strategy able to limit both the improper use of antimicrobials and the spread of multidrug-resistant pathogens.

## Figures and Tables

**Table 1:** Antimicrobials prescribed in dog and cat before the mandatory use of veterinary electronic prescription (VEP) (from April 2018 to March 2019).

Antimicrobial Classes	Antimicrobials	N° of Prescriptions in Dogs	N° of Prescriptions in Cats	N° Total	Classes %
*β*-lactams (alone or combined)	Amoxicillin/clavulanic acid	52	16	68	56.1
	Ampicillin	4		4	
	Cefadroxil	11		11	
	Cefalexin	39	6	45	
	Cefazolin	88	4	92	
	Ceftriaxone	37	2	39	
	Penicillin G/dihydrostreptomycin	9		9	
Nitroimidazoles (alone or combined)	Metronidazole	29		29	12.6
	Metronidazole/spiramycin	23	8	31	
Fluoroquinolones	Enrofloxacin	28	6	34	9.5
	Ciprofloxacin	2	1	3	
	Marbofloxacin	4		4	
	Pradofloxacin	3	1	4	
Tetracyclines	Doxycycline	21	1	22	7.3
	Oxytetracycline	12	1	13	
Aminoglycosides	Tobramycin	26	4	30	7.1
	Gentamicin	4		4	
Lincosamides	Clindamycin	9	4	13	2.7
Polymyxins	Polymyxin B	10	2	12	2.5
Sulfonamides	Sulfadiazine	1		1	1.0
	Sulfametopyrazine	3		3	
	Sulfamethoxazole	1		1	
Ansamycins	Rifampicin	2		2	0.6
	Rifaximin	1		1	
Macrolides	Azithromycin	2			0.4
Fusidanes	Fusidic acid	1		1	0.2
		422	56	478	100.0

**Table 2:** Class of antimicrobials prescribed in dogs and cats after the mandatory use of VEP (from April 2019 to March 2020).

Antimicrobial Classes	Antimicrobials	N° of Prescriptions in Dogs	N° of Prescriptions in Cats	N° Total	Classes %
*β*-lactams (alone or combined)	Amoxicillin/clavulanic acid	68	17	85	59.3
	Amoxicillin/doxycycline		1	1	
	Cefadroxil	21	3	24	
	Cefalexin	69	4	73	
	Cefalexin/clindamicin	66	2	68	
	Cefalexin/gentamicin	1		1	
	Cefalexin/enrofloxacin	1		1	
	Penicillin G/dihydrostreptomy	1		1	
Fluoroquinolones	Enrofloxacin	26	7	33	11.1
	Marbofloxacin	11	1	12	
	Orbifloxacin	2		2	
Nitroimidazoles (alone or combined)	Metronidazole	2		2	10.0
	Metronidazole/spiramycin	38	3	41	
Aminoglycosides	Gentamicin	33		33	7.7
Tetracyclines	Oxytetracyclin	2		2	3.8
	Doxycycline	13	1	14	
Lincosamides (alone or combined)	Clindamycin	6	1	7	2.1
	Clindamycin/gentamicin	1		1	
	Clindamycin/cefadroxil	1		1	
Fusidanes	Fusidic acid	8	1	9	2.1
Polymyxins	Polymyxin B	9		9	2.1
Phenicols	Florfenicol	5		5	1.1
Ansamycins/Polymyxins	Rifaximin/colistin	3		3	0.7
		387	41	428	100.0

## Data Availability

Not applicable.
